# Chronic intratracheal application of the soluble guanylyl cyclase stimulator BAY 41-8543 ameliorates experimental pulmonary hypertension

**DOI:** 10.18632/oncotarget.16769

**Published:** 2017-03-31

**Authors:** Matthieu Amirjanians, Bakytbek Egemnazarov, Akylbek Sydykov, Baktybek Kojonazarov, Ralf Brandes, Himal Luitel, Kabita Pradhan, Johannes-Peter Stasch, Gorden Redlich, Norbert Weissmann, Friedrich Grimminger, Werner Seeger, Hossein Ghofrani, Ralph Schermuly

**Affiliations:** ^1^ University of Giessen Lung Center, Giessen, Germany; ^2^ Max Planck Institute for Heart and Lung Research, Bad Nauheim, Germany; ^3^ Institute for Cardiovascular Physiology, J.W. Goethe University, Frankfurt, Germany; ^4^ Cardiology Research, Pharmaceuticals, Bayer AG, Wuppertal, Germany; ^5^ Institute of Pharmacy, Martin Luther University of Halle Wittenberg, Halle, Germany; ^6^ Research Pharmacokinetics, Pharmaceuticals, Bayer AG, Wuppertal, Germany

**Keywords:** monocrotaline, cGMP, nitric oxide, pulmonary hypertension, remodelling, Pathology Section

## Abstract

Dysfunction of the NO/sGC/cGMP signaling pathway has been implicated in the pathogenesis of pulmonary hypertension (PH). Therefore, agents stimulating cGMP synthesis *via* sGC are important therapeutic options for treatment of PH patients. An unwanted effect of this novel class of drugs is their systemic hypotensive effect. We tested the hypothesis that aerosolized intra-tracheal delivery of the sGC stimulator BAY41-8543 could diminish its systemic vasodilating effect.

Pharmacodynamics and -kinetics of BAY41-8543 after single intra-tracheal delivery was tested in healthy rats. Four weeks after a single injection of monocrotaline (MCT, 60 mg/kg s.c.), rats were randomized to a two-week treatment with either placebo, BAY 41-8543 (10 mg/kg per os (PO)) or intra-tracheal (IT) instillation (3 mg/kg or 1 mg/kg).

Circulating concentrations of the drug 10 mg/kg PO and 3 mg/kg IT were comparable. BAY 41-8543 was detected in the lung tissue and broncho-alveolar fluid after IT delivery at higher concentrations than after PO administration. Systemic arterial pressure transiently decreased after oral BAY 41-8543 and was unaffected by intratracheal instillation of the drug. PO 10 mg/kg and IT 3 mg/kg regimens partially reversed pulmonary hypertension and improved heart function in MCT-injected rats. Minor efficacy was noted in rats treated IT with 1 mg/kg. The degree of pulmonary vascular remodeling was largely reversed in all treatment groups.

Intratracheal administration of BAY 41-8543 reverses PAH and vascular structural remodeling in MCT-treated rats. Local lung delivery is not associated with systemic blood pressure lowering and represents thus a further development of PH treatment with sGC stimulators.

## INTRODUCTION

Despite recent achievements in treatments [1], pulmonary arterial hypertension (PAH) remains a devastating disease with a poor prognosis [2]. Recently, a new class of drugs has been proposed for treatment of the disease - soluble guanylate cyclase (sGC) stimulators.

Stimulation of sGC by nitric oxide results in an enhanced production of the second messenger cGMP, which regulates a plethora of physiologic processes including vascular tone, fibrosis, proliferation, inflammation, microvascular permeability and neutrophil-endothelium interactions [3]. Chronic treatment with the sGC stimulator BAY 41-2272 has been demonstrated to reverse hemodynamic and structural changes associated with monocrotaline (MCT)- and chronic hypoxia-induced experimental pulmonary hypertension (PH) suggesting a strong anti-remodeling potency of this class of drugs [4]. In accordance with these findings, BAY 41-2272 demonstrated anti-proliferative effects on vascular smooth muscle cells [5]. Thus, the new class of drugs targets two key processes involved in the pathogenesis of PAH: vasoconstriction and vessel remodeling. The sGC stimulators (BAY 41-2272 and BAY 41-8543) were less successful due to low efficacy and low oral bioavailability [6, 7]. Riociguat (BAY 63-2521) is the first sGC stimulator that has made a successful transition from animal experiments to controlled clinical studies in patients. Riociguat showed favorable drug metabolism and pharmacokinetic profiles [8]. In different experimental models of pulmonary hypertension, riociguat had beneficial effects on pulmonary haemodynamics, right heart hypertrophy and remodelling of the pulmonary vasculature [9]. In phase III clinical trials, riociguat demonstrated efficacy in patients with PAH and, remarkably, also in patients with chronic thromboembolic pulmonary hypertension (CTEPH) [10, 11]. Recently, riociguat (Adempas^®^) was approved by the Food and Drug Administration (FDA) and European Medicines Agency for the treatment of these two forms of pulmonary hypertension: inoperable, recurrent or persistent CTEPH and PAH [12].

Experiments in animal models of PH have revealed a strong systemic vasodilating effect of sCG stimulators in lambs [13], mice and rats [14]. Even in patients, a decrease in systemic arterial pressure was reported as a side effect of riociguat [15].

A possible way to overcome the systemic vasodilating effect would be a local delivery of the drug into the lung. In fact, inhaled agonists of sGC induced selective pulmonary vasodilation in a model of acute pulmonary vasoconstriction [16]. The present study was designed to investigate the question whether a long term intra-tracheal administration of BAY 41-8543 would be therapeutically efficient in a model of chronic PAH while remaining a selective vasodilator for pulmonary vasculature. The pharmacokinetic profile of the drug was investigated after peroral (PO) and intra-tracheal (IT) delivery. We examined acute and chronic hemodynamic effects of intra-tracheal BAY 41-8543, and anti-remodeling effects of the long-term administration of this agent.

## RESULTS

### Pharmacokinetics and tissue distribution of BAY 41-8543 after oral and intratracheal administration

**Figure 1:**
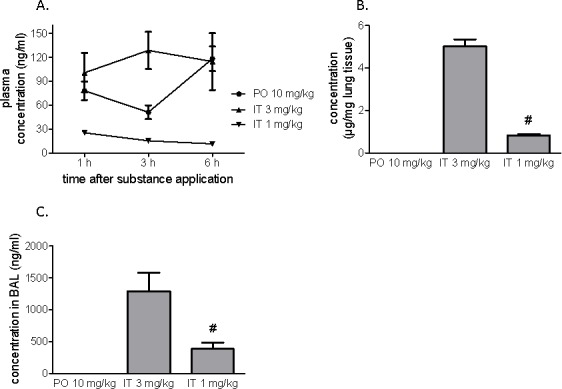
Pharmacokinetics after a single application of BAY 41-8543 The substance was administered either orally or intratracheally. A. Plasma concentrations of the substance one, three and six hours after administration. **B**. Concentrations of the substance in the lung tissue six hours after a single administration. **C**. Concentrations of the substance in the broncho-alveolar lavage fluid performed (BAL) six hours after administration. Data presented as mean ± SEM. # *p* < 0.05 IT 1 mg/kg *vs*. IT 3 mg/kg group.

Peroral administration of 10 mg/kg of BAY 41-8543 resulted in the highest plasma concentrations 6 h post-application (Figure 1A). In contrast, intra-tracheal administration of 3 mg/kg provided stable plasma concentration in magnitude comparable with that of peroral administration (at 6h - 114.6 ± 31.9 ng/ml). The IT dose of 1 mg/kg resulted in lower plasma concentrations of the substance (at 6h - 11.5 ± 0.8 ng/mL).

In the lung tissue samples, IT 3 mg/kg produced a local substance concentration of 5.0 ± 0.3 μg/mg lung tissue, IT 1 mg/kg - 0.8 ± 0.1 μg/mg lung tissue, whereas PO 10 mg/kg produced a 3 orders of magnitude lower concentration of 1.0 ± 0.2 ng/mg lung tissue (Figure 1B).

Six hours after intratracheal application, the compound was detected in BAL fluid where IT 3 mg/kg demonstrated 3-fold higher concentration than IT 1 mg/kg. After peroral administration, the substance concentration in BAL fluid was below the limit of quantification of the assay (Figure 1C).

### Intratracheally administered BAY 41-8543 has no effect on systemic arterial pressure

**Figure 2:**
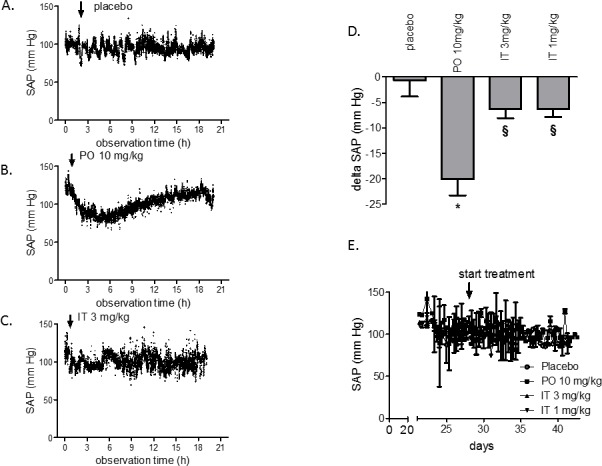
Acute and chronic effects of BAY 41-8543 on systemic arterial pressure in monocrotaline model of PAH Four weeks after monocrotaline injection, treatment with BAY 41-8543 was started. Representative tracings of the systemic arterial pressure (SAP) monitoring are shown after **A**. per-oral placebo treatment, **B**. per-oral treatment with 10 mg/kg of BAY 41-8543, **C**. intra-tracheal treatment with 3 mg/kg of BAY 41-8543. **D**. Quantification of the systemic hypotensive effect after a single substance administration is demonstrated. Data presented as delta between SAP values before and 4 hours after drug administration. **E**. Results of SAP monitoring twice daily in rats treated chronically either per-oral or intra-tracheally. Data presented as mean ± SEM. * *p* < 0.05 PO 10 mg/kg *vs*. placebo, § *p* < 0.05 IT *vs*. PO 10 mg/kg group.

None of the manipulations (PO or IT application) induced changes in SAP (Figure 2). Peroral administration of 10 mg/kg of BAY 41-8543 to rats induced a decrease in SAP by 27 mmHg (Figure 2A) lasting for at least 5 hours as shown on the representative tracing of pressure recordings. In contrast, SAP was only slightly affected in rats after IT instillation of the agent (Figure 2D). Notably, chronic PO treatment with BAY 41-8543 did not result in accumulation of its systemic hypotensive effect, as chronic SAP measurements twice daily mornings and evenings revealed similar pressures in PO and IT groups (Figure 4E).

**Figure 3:**
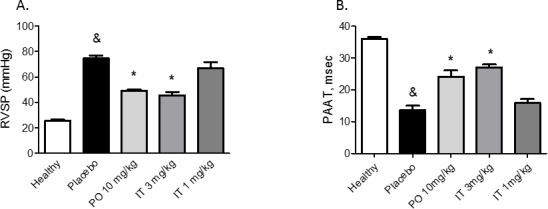

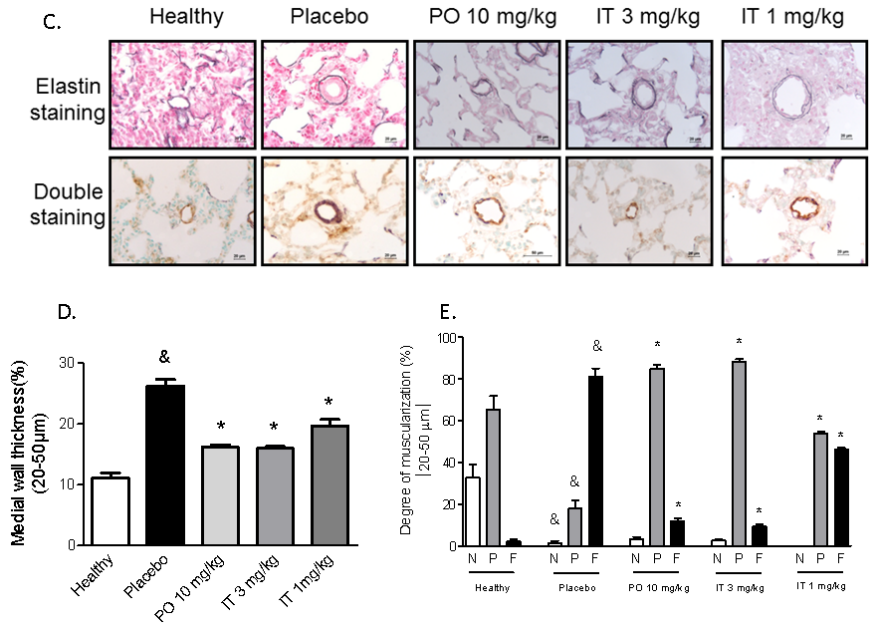
Effects of chronic treatment with BAY 41-8543 on right ventricular systolic pressure and vessel remodeling **A**. Right ventricular systolic pressure (RVSP) was measured invasively by right heart catheterization after two weeks of treatment. **B**. Pulmonary artery acceleration time (PAAT) was measured by echocardiography after two weeks of treatment. PAAT was measured from the pulsed-wave Doppler flow velocity profile of the right ventricular outflow tract in the parasternal short-axis view and defined as the interval from the onset to the maximal velocity of forward flow. **C**. Representative photomicrographs of elastin and double stained lung sections are demonstrated. **D**. Quantification of the medial wall thickness based on elastin staining. Medial wall thickness was defined as the distance between the lamina elastica interna and the lamina elastica externa. **E**. Quantification of the degree of vessel wall muscularisation based on double staining. Arteries that contained > 70% of α-actin positive-vessel wall area were set as fully muscularized (on the figure labelled as F); arteries with < 4% of α-actin positive-vessel area were set as non-muscular (on the figure labelled as N). Arteries that contained 4-70% of α-actin positive-vessel area were defined as partially muscularized (on the figure labelled as P). Data presented as mean ± SEM. & *p* < 0.05 placebo *vs*. healthy, * *p* < 0.05 treatment groups *vs*. placebo.

**Figure 4:**
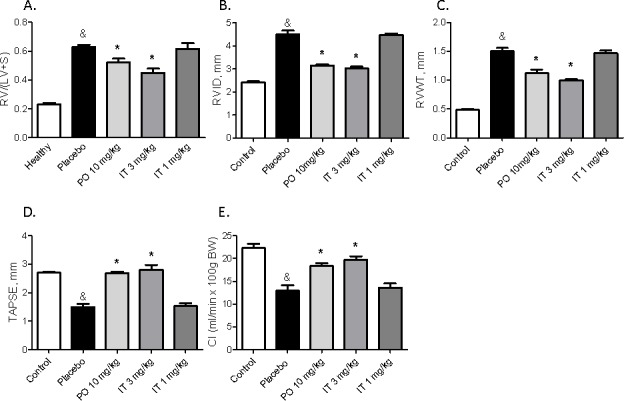

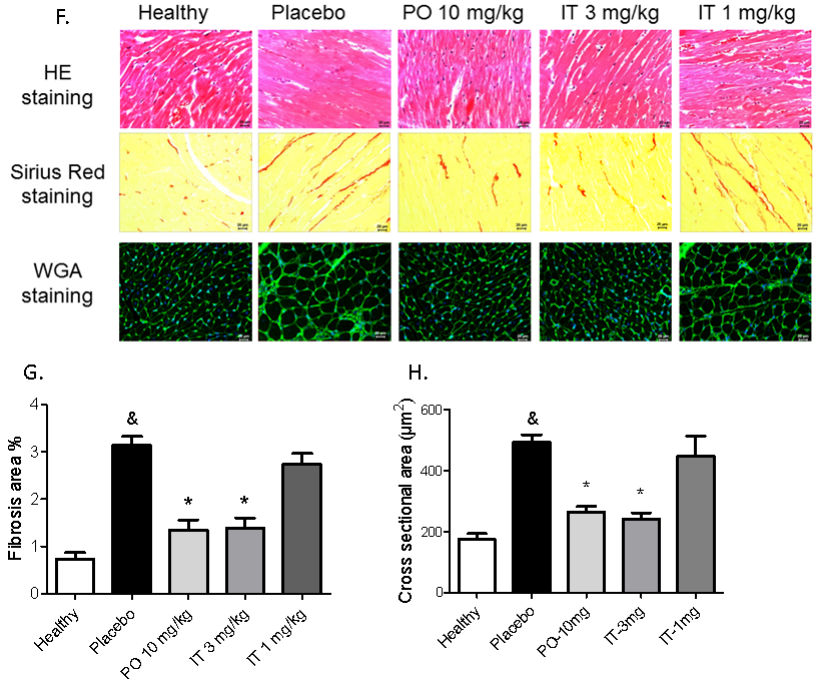
Effects of chronic treatment with BAY 41-8543 on the right ventricular remodelling and function **A**. The heart was dissected and the ratio of the right ventricle weight to left ventricle plus septum weight (RV/LV+S) was calculated. **B**. Right ventricular internal diameter (RVID), **C**. Right ventricular free wall thickness (RVWT), and **D**. Tricuspid annular plane systolic excursion (TAPSE) were measured by echocardiography. **E**. Cardiac output was calculated using the Fick’s principle and normalized to body weight to obtain cardiac index (CI). **F**. Representative images of sirius red staining for collagen visualization and WGA staining for cardiomyocyte size measurement from different groups are demonstrated. **G**. Results of quantification of the sirius red stained heart sections are demonstrated. **H**. Results of cardiomyocyte cross sectional area quantification are demonstrated. Data presented as mean ± SEM. * *p* < 0.05 placebo *vs*. healthy, & *p* < 0.05 placebo *vs*. healthy, * *p* < 0.05 treatment groups *vs*. placebo.

### Intratracheal BAY 41-8543 attenuates the development of pulmonary hypertension and pulmonary vascular remodeling

Twenty-eight days after injection of monocrotaline (MCT) (placebo group), severe PAH developed with a marked increase in RVSP and a decrease in PAAT (Figure 3). Both, PO treatment with 10 mg/kg and IT treatment with 3 mg/kg of BAY 41-8543 significantly decreased RVSP and increased PAAT. There was a non-significant improvement in RVSP after IT instillation of 1 mg/kg of BAY 41-8543.

Injection of MCT resulted in medial hypertrophy and a pronounced increase in muscularization of the distal pulmonary arteries (20-50 μm in diameter) after 28 days compared with pulmonary arteries from control saline-injected rats (Figure 3A-3C). Treatment with BAY 41-8543 both PO and IT at the dose of 3 mg/kg significantly reduced the number of fully muscularized pulmonary arteries and resulted in an increase of partially muscularized pulmonary arteries compared with the MCT-placebo group (Figure 3A-3C). Intratracheal administration of 1 mg/kg of BAY 41-8543 resulted in partial reduction of pulmonary vascular remodeling (Figure 3A-3C).

### Intratracheal BAY 41-8543 improves RV remodeling and heart function

Heart remodeling of MCT rats (placebo group) was reflected by RV hypertrophy and RV dilation (Figure 4A-4C). Both, the hypertrophy and the chamber dilation were ameliorated by treatment with 10 mg/kg of BAY 41-8543 PO and 3 mg/kg IT (Figure 4A-4C).

Echocardiography revealed impaired RV function in placebo-treated rats, which was reflected by decreased TAPSE and reduced cardiac index (Figure 4D and 4E). Treatment with 10 mg/kg of BAY 41-8543 PO as well as 3 mg/kg IT resulted in significant improvement of RV remodeling and function (Figure 4A-4C). Treatment with 1 mg/kg of BAY 41-8543 IT had no effects on all of these parameters (Figure 4).

MCT-induced PH resulted in cardiac remodeling characterized by development of myocardial fibrosis and myocyte hypertrophy. Both, fibrosis and myocyte hypertrophy were suppressed by treatment with 10 mg/kg of BAY 41-8543 PO and 3 mg/kg IT (Figure 4F-4H). In contrast, IT treatment with 1 mg/kg of BAY 41-8543 had only limited impact on all of these parameters.

### Circulating and lung tissue concentrations of BAY 41-8543 after chronic treatment

**Figure 5:**
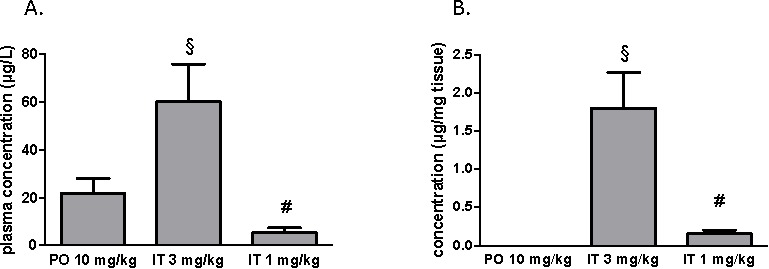
Measurements of BAY 41-8543 concentrations **A**. in plasma and **B**. in the lung tissue after chronic treatment. § *p* < 0.05 IT 3 mg/kg *vs*. PO 10 mg/kg, # *p* < 0.05 IT 1 mg/kg *vs*. IT 3 mg/kg.

After two weeks of treatment with BAY 41-8543, plasma and tissue concentrations of the substance were measured. Sample collection was performed at least 12 hours after the last drug application. In the PO treatment group, the substance plasma concentration of 22.0 ± 6.1 μg/L was measured (Figure 5A). Interestingly, IT treatment with 3 mg/kg resulted in a significantly higher plasma concentration. Intratracheal treatment with 1 mg/kg produced the substance plasma concentration of 5.3 ± 2.0 μg/L. After chronic PO treatment, no measurable levels of BAY 41-8543 were detected in lung tissue, whereas IT drug application dose dependently generated a local depot of the substance (Figure 5B).

## DISCUSSION

Inhalation offers great potential for rapid delivery, less degradation by drug-metabolizing enzymes, and high bioavailability of small-molecule therapeutics [17]. In addition, intrapulmonary administration of drugs in the therapy of PAH provides selectivity of the hemodynamic effects to the lung vasculature and reduces systemic effects [18]. Prolonged retention of some hydrophobic drugs in lung tissues has been demonstrated, explaining the extended duration of effects of those drugs [19]. Against this background, we sought to compare the effects of peroral and intra-tracheal drug delivery. The key findings of this study are as follows: 1. Chronic intra-tracheal delivery of BAY 41-8543 could be as efficient as peroral; 2. Pulmonary selectivity of the vasodilation could be achieved by the IT delivery of the drug.

### Acute pharmacokinetics and pharmacodynamics

Peroral BAY 41-8543 administration resulted in the highest plasma concentrations 6 hours post-application. Large t -values at high dosages are not uncommon. The slightly lower concentration at 3 hours *vs*. 1 hour was observed suggestive of double peak pharmacokinetics. For the lipophilic substances, like BAY 41-8543, a possible mechanism of the phenomenon could be the enterohepatic recycling [20]. On the other hand, these data should not be overinterpreted since only 3 concentration/time data points were analyzed. Intra-tracheal administration of 3 mg/kg provided a stable plasma concentration in the magnitude comparable with that of peroral administration. A potential explanation for the observed effect could be that IT administration results in a better bioavailability of the drug than the PO one [7]. The dosage IT 1 mg/kg resulted in lower plasma concentrations of the substance. There is a hint for a non-linear increase of exposure between 1 and 3 mg/kg IT. Intra-tracheal administration generated a local depot of the substance. In the lung tissue samples, IT 3 mg/kg produced a local substance concentration of 5.0 ± 0.3 μg/mg lung tissue, IT 1 mg/kg - 0.8 ± 0.1 μg/mg lung tissue, whereas PO 10 mg/kg produced a 3 orders of magnitude lower concentration of 1.0 ± 0.2 ng/mg lung tissue. Thus, intra-tracheal delivery of BAY 41-8543 generates a local depot of the substance with constant release into circulation.

### Acute and chronic effects of BAY 41-8543 on systemic arterial pressure

Systemic hypotension is a consequence of compromised hemodynamics and is a frequent condition in PAH patients. In clinical studies, a dose dependent systemic blood pressure lowering effect of riociguat in PAH patients was observed [21]. This pressure decrease can be critical in PAH patients by exacerbating existing hypotension and causing syncope. Indeed, hypotension developed in 10% of patients receiving the highest tolerated dose of riociguat [10]. Potentially, local administration of the substance could restrict the vasodilating effect to the lung vasculature and avoid systemic side effects. Indeed, a transient decrease in SAP was observed after orally given 10 mg/kg of BAY 41-8543 in rats in our study. On the contrary, lowering of the SAP was only marginal after IT application of the drug. These findings are in line with the results of Evgenov et al., who demonstrated selective pulmonary vasodilating effects of inhaled sGC stimulators in the setting of acute PH [16]. Thus, inhaled delivery of the drug diminished systemic side effect of the substance. Interestingly, while the effects of PO 10 mg/kg and IT 3 mg/kg on the systemic pressure are different, plasma concentrations of the substance were comparable after single administrations. The most probable explanation for this discrepancy is that the acute blood pressure lowering effect is not only dependent on the concentration of the drug in plasma but also on its local tissue concentration. The arcade arteries of the mesenteric vascular bed have been demonstrated to contribute substantially to the control of peripheral resistance [22]. Indeed, perindopril injection reduced the systemic blood pressure and to a greater extent the pressure in the arcade arteries [23]. Potentially, orally delivered vasoactive substances could build a tissue depot in the gastro-intestinal tract and induce a preferential vasodilation of the mesenteric arteries causing systemic hypotension. In line with this, Sramek et al. have shown that a 125 mg of intravenous (i.v.) captopril dose (a 10-fold higher dose) was not more effective in blood pressure reduction than a 12.5 mg i.v. or a 25 mg PO dose [24].

One of the most frequently reported side effects of sGC stimulators riociguat is systemic hypotension [11, 25]. As next, we sought to test whether chronic peroral treatment with BAY 41-8543 could lead to accumulation of its systemic hypotensive effect. For this purpose, SAP was measured in chronically treated rats twice daily: mornings - before drug administration and evenings - eight to ten hours after drug administration. No significant difference in SAP was observed between PO and placebo groups. As expected, no systemic hypotension was observed in IT groups. Thus, both PO and IT treatment regimens are safe and do not induce accumulation of the hypotensive effect with chronic drug administration.

Recent studies suggest that concomitant administration of agents from two or more classes of drugs is more effective in PAH patients [26]. Riociguat can be used in combination with endothelin receptor antagonists or prostanoids [10, 27]. However, combination of riociguat with a standard dose of sildenafil showed no evidence of a positive risk-benefit assessment, predominantly because of a high rate of discontinuation due to hypotension in long-term extension [28]. Our results indicate that IT aerosolized delivery for this class of drugs could be an alternative approach to limit its systemic effects. It is tempting to speculate that the observed effect provides a rationale for concomitant administration of aerosolized formulations of sGC stimulators and other orally administered vasoactive drugs for treatment of PAH to enhance the pulmonary hypotensive effect while avoiding systemic hypotension. In this regard, combination of aerosolized formulations of sGC stimulators with phosphodiesterase-5 inhibitors may reduce the rate of discontinuation due to hypotension. This hypothesis warrants further investigations.

### Chronic effects of BAY 41-8543 on lung vasculature and heart remodeling

We have previously shown anti-remodeling effects on pulmonary vasculature after chronic treatment with sGC stimulators [4, 29, 30]. In the current study, we found that chronic treatment with BAY 41-8543 both PO and IT at a dose of 3 mg/kg significantly reduced RVSP and reversed vascular remodeling of the lung vasculature. The most dangerous consequence of PAH is an impaired cardiac function, which is a predictor of poor outcome [2]. We observed significantly remodeled RV and impaired cardiac function in the placebo-treated MCT-rats. RV hypertrophy was reversed and heart function improved in MCT-rats that were treated with 10 mg/kg of BAY 41-8543 PO or 3 mg/kg IT. Cardiomyocyte enlargement and increased interstitial fibrosis were present in MCT-rats treated with placebo and were reversed by treatment with BAY 41-8543 given PO at a dose of 10 mg/kg or IT at a dose of 3 mg/kg.

In the groups, PO 10 mg/kg and IT 3 mg/kg, reversal of RV hypertrophy and fibrosis can be explained by hemodynamic unloading of the RV and probably stimulation of the cGMP-signaling in the heart. Reduction of the RV afterload can induce reversal of cardiac remodeling as it has been demonstrated in patients with PAH after isolated lung transplantation [31]. A direct effect of the circulating substance is possible as plasma concentrations of BAY 41-8543 were comparable between the PO 10 mg/kg and IT 3 mg/kg groups. Anti-fibrotic effect of pharmacological sGC stimulation on fibrosis in pressure-overloaded rat heart has been reported [32]. Anti-hypertrophic actions of sGC stimulators in the models of left ventricular hypertrophy have also been demonstrated [33–35].

Chronic IT treatment in a dose of 1 mg/kg reduced partially the degree of lung vascular remodeling as reflected by decrease in medial wall thickness and in the number of fully muscularized vessels. However, these changes were insufficient to reduce RVSP. No effects on cardiac remodeling were detected in IT 1 mg/kg group. Probably, insufficient hemodynamic unloading and lower circulating levels of the substance are responsible for the absence of cardiac effects of the drug.

The advantage of the aerosolized drug delivery is the possibility to generate locally high concentrations in the lung. Indeed, significant levels of some drugs can still be measured in the lungs up to 40 hours after IT dosing [36]. Furthermore, very long duration of action has been shown even for some drugs with a short half-life after inhalative administration [37]. We interpret the results that the long duration of action of our compound is due to persistence of the active compound in the lung tissue. Intra-tracheal drug delivery generates a tissue depot of the substance distributed in different cell types, interstitial space, and alveolar lining fluid. Therefore, the substance concentration measurements from the whole tissue lysate might not reflect the concentration of the substance in the vascular smooth muscle cells, which is the efficient concentration in the effector cells. Moreover, absorption of therapeutic agents from the lungs can be prolonged by a variety of mechanisms so that the effect can persist over longer time thus decreasing the need for repeated administrations of the drug [17].

### Study limitations

IT instillation of the substance in the surfactant solution was used in our study with assumption that this approach is similar to aerosolization. Potentially, our approach could result in unequal drug deposition and localized area of fluid overload. However, our control experiments with dye delivery using the same vehicle have demonstrated ubiquitous distribution of the dye throughout the entire lung. Microscopic evaluation of the lungs did not reveal any areas of pneumonia or atelectasis.

The MCT-induced PAH model used in our study is often criticized because it does not fully mimic the histopathology of the human disease [38]. Nevertheless, the model recapitulates most of the relevant features of the disease relevant to our study: pronounced increase in lung vascular resistance, cardiac dysfunction, and decrease in cardiac output.

### Summary

Thus, chronic intra-tracheal administration of BAY 41-8543 reverses PAH and vascular structural remodeling in MCT-treated rats. This regimen is not associated with systemic hypotension, a significant side effect of oral sGC stimulators. Our findings demonstrate a potential for development of a new therapeutic approach for treatment of PAH by inhalative administration of sGC stimulators, which could provide pulmonary selectivity of hemodynamic effects, and reduce systemic side effects.

## MATERIALS AND METHODS

### Animals

Adult Sprague Dawley rats (300-350 g body weight) were obtained from Charles River Laboratories (Sulzfeld, Germany). Local authorities approved the study protocol.

### Acute pharmacokinetic study

For the acute pharmacokinetic study, rats received one of the following treatments: a) PO placebo (400 μL of 2% methylcellulose solution) by gavage (*n* = 5); b) IT placebo (400 μL of 0.2% surfactant solution) by IT instillation (*n* = 5); c) BAY 41-8543 (10 mg/kg) PO by gavage (*n* = 5); d) BAY 41-8543 (3 mg/kg) by IT instillation (*n* = 5); e) BAY 41-8543 (1 mg/kg) by IT instillation (*n* = 5). One, three, and six hours after treatment, blood samples were collected from the vena saphenous under anaesthesia with 3% isoflurane. Plasma was obtained from the blood samples by centrifugation at 4°C for 10 min at 1000g. Afterwards, rats were sacrificed and bronchoalveolar lavage (BAL) was performed with 10 mL of isotonic saline solution. The BAL fluid (BALF) was immediately frozen and stored at −80°C. After midsternal thoracotomy pulmonary artery was canulated and flushed with 15 mL of isotonic saline. Afterwards, the lung tissue was harvested, immediately frozen, and stored at −80°C.

### Chronic treatment study

PAH was induced in adult Sprague-Dawley rats by MCT injection as described previously in detail [39]. Briefly, MCT (Sigma, Deishofen, Germany) was administered as a single subcutaneous injection at a dose of 60 mg/kg body weight. After 28 days, rats were randomized into the following treatment groups:

- PO placebo - treated once daily orally with 400 μL of vehicle (2% methylcellulose solution) by gavage for 14 days (*n* = 10);
- IT placebo - treated every second day with 400 μL of vehicle (0.2% surfactant solution) by IT instillation for 14 days (*n* = 10);
- PO 10 mg/kg - treated once daily orally with BAY 41-8543 (10 mg/kg) by gavage daily for 14 days (*n* = 10);
- IT 3 mg/kg - treated every second day with BAY 41-8543 (3 mg/kg) by IT instillation for 14 days (*n* = 10);
- IT 1 mg/kg - treated every second day with BAY 41-8543 (1 mg/kg) by IT instillation for 14 days (*n* = 10).

As oral and IT placebo groups were not different from each other, their results were pooled and presented as one placebo group. BAY 41-8543 was synthesized in the laboratories of Bayer Pharma AG (Germany). The 10 mg/kg daily dose of BAY 41-8543 was chosen based on previous studies [4]. The dose for IT delivery was selected based on the previously described experience in transition from PO to aerosolized drug delivery [40].

### Intratracheal instillation

Oral endotracheal intubation of rats was performed under isoflurane anaesthesia and then the active compound or vehicle was instilled into the trachea under direct visual control, as previously described [41]. Afterwards, rats were allowed to recover while breathing 100% oxygen.

### Radiotelemetry

Systemic arterial pressure (SAP) was monitored by an implantable telemetry system as described previously [39]. PhysioTel^®^ PA-C40 small animal pressure transmitters (DSI International, Tilburg, Netherlands) were implanted into the femoral artery under anesthesia (See online supplement for details). Animals were allowed to recover and were housed individually in standard rat cages. The pressure signal was transferred to a remote receiver (model RPC-1) and a data-exchange matrix connected to a computer. After surgery, rats were allowed to recover for 3 days. The SAP stabilized in the first 24 hours. None of the animals manifested signs of inflammation or infection. Pressure measurements were started 30 min before drug administration and continued for 24 hours thereafter.

### Hemodynamic and RV hypertrophy measurements

Right ventricular systolic pressure (RVSP) was measured by a catheter inserted into the RV *via* the right jugular vein, and for SAP the left carotid artery catheterization was performed as described [39]. The heart was dissected to separate the RV from the left ventricle plus septum (LV+S), and the ratio RV/(LV+S) was calculated as a measure of right ventricular hypertrophy (See online supplement for details).

### Histology and pulmonary vascular morphometry

Lung tissue preparation, sectioning, staining, and vascular morphometry were done as described [39]. Intraacinar arteries were analysed by categorizing them as muscular, partially muscular, and nonmuscular. In addition, the medial wall thickness of the vessels was analysed. All analyses were done in a blinded fashion (See online supplement for details).

### Measurement of the BAY 41-8543 concentration in plasma, BALF, and lung tissue samples

For pharmacokinetics study, the plasma samples, lung tissue, and BALF samples were collected at indicated time points (see section “Acute pharmacokinetic study”). For measurements after chronic treatment, plasma and lung tissue samples were collected next morning after the last drug administration corresponding to 18-24 hours post-administration.

The lung tissue was lyophilized and homogenized. Lung tissue, BALF and plasma samples were treated with acetonitril and ammoniumacetate-buffer (0.01 M, pH 6.8). After centrifugation at 1000 g the supernatant was subjected to LC-MS/MS for quantification of BAY 41-8543. Therefore, an Agilent 1100 HPLC with a flow rate of 400 μL/min was used. A linear gradient on a Luna 5 μm C8(2) 100A 50×2 mm (Phenomenex) separation column from 10 to 85% of acetonitrile with 0.1% formic acid was performed against a second mobile phase of ammoniumacetate-buffer (0.01 M, pH 6.8). A TurboV ionsource was used to transfer the eluate into the API4000 (AB Sciex) mass spectrometer. The lower limit of quantification was 1 μg/L.

### High-resolution echocardiography

Echocardiographic measurements were performed in isoflurane anesthetized spontaneously breathing rats using VisualSonics Vevo770 high-resolution imaging system equipped with a 25-MHz transducer (VisualSonics, Toronto, Canada) as described [42] with modifications (See online supplement for details).

### Heart tissue histology

Paraffin-embedded right ventricular sections were stained with wheat germ agglutinin or picrosirius red to determine cardiomyocyte size and interstitial fibrosis. Photomicrographs were quantified to determine the mean cross-sectional area of cardiomyocytes and collagen content using the Leica Qwin V3 computer-assisted image analysis software (Leica Microsystem, Wetzlar, Germany) (See online supplement for details).

### Data analysis

All data are presented as mean ± SEM. First, the data from normal rats were compared with data from the monocrotaline group (the disease model), using an unpaired *t*-test. Following this, the placebo and treatment groups were analysed by ANOVA with following Student Newman-Keuls *post hoc* test for multiple comparisons. A value of *p* < 0.05 was considered significant.

## SUPPLEMENTARY MATERIALS


