# Supplementary material for: Chronic intratracheal application of the soluble guanylyl cyclase stimulator BAY 41-8543 ameliorates experimental pulmonary hypertension
Source: Oncotarget. 2017 Mar 31;8(18):29613–24. doi: 10.18632/oncotarget.16769 (PMC5444690; doi:10.18632/oncotarget.16769)
Supplement: Supplementary file 1 [file oncotarget-08-29613-s001.pdf]

Chronic intratracheal application of the soluble guanylyl cyclase stimulator BAY 41-8543 ameliorates experimental pulmonary hypertension

Supplementary Material

Reagents

All reagents were obtained from Sigma-Aldrich (Munich, Germany) in not otherwise stated. BAY 41-8543 was provided by Bayer HealthCare (Wuppertal, Germany).

Acute pharmacokinetic study

For the acute pharmacokinetic study, rats received one of the following treatments: a) oral placebo (400 µl of 2 % methylcellulose solution) by gavage (n = 5); b) intratracheal placebo (400 µl of 0.2 % surfactant solution) by intratracheal instillation (n = 5); c) BAY 41-8543 (10 mg/kg) orally by gavage (n = 5); d) BAY 41-8543 (3 mg/kg) by intratracheal instillation (n = 5); e) BAY 41-8543 (1 mg/kg) by intratracheal instillation (n = 5). One, three, and six hours after treatment, blood samples were collected from vena saphenous under anaesthesia with 3% isoflurane. Plasma was obtained from the blood samples by centrifugation at 4°C 10 min 1000g. Afterwards, rats were sacrificed and bronchoalveolar lavage (BAL) was performed with 10 ml of isotonic saline solution. BAL fluid (BALF) was immediately frozen and stored at -80 °C. After misternal thoracotomy pulmonary artery was canulated and flushed with 15 ml of isotonic saline. Afterwards, the lung tissue was harvested, immediately frozen, and stored at -80 °C.

Pressure transmitter implantation. Under anesthesia with isoflurane 3-5%, rats were intubated and ventilated using small animal ventilator with frequency 60 min^{-1} and 150 ml minute ventilation. Skin of the antero-medial surface of the rat's right leg was shaved and wet with Braunoderm. After cutting skin, subcutane tissue was prepared and femoral artery was prepared free from surrounding tissue. PhysioTel® PA-C40 small animal pressure transmitter (DSI International, Tilburg, Netherlands)

was implanted into femoral artery and fixed with ligatures. The body of the transmitter was placed in the cavity generated in the lateral abdominal subcutaneous connective tissue. Subcutaneous tissue and skin was closed with two series of sutures. To release the post-operative pain, Buprenorphine 50 µg/kg and Indometacine 20 mg/kg was injected subcutaneously. Animals were allowed to recover under heating lamp breathing 50 % oxygen and were housed individually in standard rat cages. The pressure signal was transferred to a remote receiver (model RPC-1) and a data-exchange matrix connected to a computer. After surgery, rats were allowed to recover for 3 days. The SAP stabilized in the first 24 hours. None of the animals manifested signs of inflammation or infection. Pressure measurements were done 30 min before drug administration and continued for 24 hours thereafter.

MCT treatment

Monocrotaline (Sigma, Deishofen, Germany) was dissolved in 1 M HCl, adjusted to pH 7.4 with 1 M NaOH, sterilized by passing through Minisart sterile filter (Sartorius, Göttingen, Germany) 0.2 µm, and administered as a single subcutaneous injection at the dose of 60 mg/kg body weight, as described [31]. Control rats received an equal volume of isotonic saline.

Hemodynamic and right ventricular hypertrophy measurements

For measurement of hemodynamic parameters, rats were anaesthetised with intra-peritoneal injection of ketamine (9 mg/kg body weight) and medetomidine (100 µg/kg body weight). Afterwards, rats received intramuscular injection of atropine (250 µg/kg body weight) to minimise vasovagal side effects during the preparation. The rats were tracheotomised and artificially ventilated at a constant frequence of 60 breaths per minute with inspiratory flow rate of 500-600 cc/min. Positive end-expiratory pressure was set at 1 cmH_2O. The left carotid artery was isolated and cannulated with a polyethylene tubing connected to a fluid-filled force transducer and the systemic arterial pressure (SAP) was measured. A catheter was inserted through the right jugular vein into the right ventricle for measurement of right ventricular systolic pressure (RVSP). Cardiac output was calculated using the Fick's principle, by employing the mixed venous oxygen and the arterial oxygen content as previously described (Schermuly *Am J Respir Crit Care Med* 160:1500-1506). Arterial and mixed venous samples were collected (150 µL) and analyzed for partial pressure of

oxygen, pH and carbon dioxide tension, haemoglobin and oxygen saturation (Rapidlab 348, Bayer Diagnostics, Leverkusen, Germany). Pulmonary vascular resistance indexed to the body weight (PVRI) was determined as described previously (Schermuly *Circulation* 115:2331-2339). The animals were ex-sanguinated and the lungs were flushed with sterile saline to get rid of blood. The left lung was fixed for histology in 3.5 % neutral buffered formalin and the right lung was snap frozen in liquid nitrogen. The ventricles were dissected free of the great vessels and atria. The right ventricle (RV) was separated from the left ventricle and ventricular septum (LV + S). The RV and (LV + S) were patted dry and weighed. We evaluated right ventricular hypertrophy using the ratio of RV to (LV + S).

High-resolution echocardiography

Anesthesia was induced with 3% isoflurane gas and maintained with 1.0 - 1.5% isoflurane in 100% O_2. Rats were laid supine on a heating platform with all legs taped to ECG electrodes for heart rate (HR) monitoring. Body temperature was monitored via a rectal thermometer (Indus Instruments, Houston, TX) and maintained at 36.5-37.5°C using a heating pad and an infrared lamp. The chest of the rats was shaved and treated with a chemical hair remover to reduce ultrasound attenuation. To provide a coupling medium for the transducer, a pre-warmed ultrasound gel was spread over the chest wall.

Transthoracic two-dimensional, M-mode and Doppler imaging were performed with VisualSonics Vevo770 high-resolution imaging system equipped by 25-MHz transducer (VisualSonics, Toronto, Canada) 2 weeks after treatment and 24 h before catheterisation study. RV free wall thickness (RVWT) was measured in the modified parasternal long-axis view. RV outflow tract (RVOT) dimension was measured from the RVOT view at the level of the aortic valve. For determination of TAPSE M-mode cursor was oriented to the junction of the tricuspid valve plane with the RV free wall using the apical four chamber view. Pulmonary artery acceleration time (PAAT) was measured from the pulsed-wave Doppler flow velocity profile of the RV outflow tract in the parasternal short-axis view and defined as the interval from the onset to the maximal velocity of forward flow. All echocardiographic parameters were calculated off-line using tool section of the Visual Sonics Vevo770 system. All the studies were performed by an experienced sonographer who was blinded to results of invasive and morphometric studies.

Histology and pulmonary vascular morphometry

The formalin-fixed lungs were subjected to paraffin embedding. The paraffin-embedded tissues were subject to sectioning to yield 3 μm thick sections. Elastica staining was performed according to common histopathological procedures. The degree of muscularization of small peripheral pulmonary arteries was assessed by double-staining the 3 μm sections with an anti-α-smooth muscle actin antibody (dilution 1:900, clone 1A4, Sigma, Saint Louis, Missouri) and antihuman von Willebrand factor antibody (vWF, dilution 1:900, Dako, Hamburg, Germany) followed by analysis of the vessels using a computerized morphometric analysis system (QWin; Leica, Wetzlar, Germany). In each rat, 80 to 100 intra-acinar arteries (25 to 50 μm diameter) were categorized as muscular, partially muscular, or non-muscular. Arteries of the same size were additionally analyzed for the medial wall thickness as previously described (Schermuly *J Clin.Invest* 115:2811-2821). All analyses were done in a blinded fashion.

Heart tissue histology

Freshly dissected RV tissue was fixed in 4% paraformaldehyde overnight, then dehydrated and embedded in paraffin and sectioned at a thickness of 3 μm. For cardiomyocyte size determination sections were stained with FITC conjugated wheat germ agglutinin (WGA-FITC, Sigma-Aldrich, Steinheim, Germany). Nuclei were stained with 4',6-diamidino-2-phenylindole (DAPI, Invitrogen, Karlsruhe, Germany) and mounted in DAKO fluorescent mounting medium (DAKO Diagnostika, Hamburg, Germany). Sections without WGA-FITC and DAPI were used as a negative control. To detect collagen fibers RV sections were stained with 0.1% Sirius Red (Sirius Red F3B, Niepoetter, Bürstadt, Germany) in picric acid (Fluka, Neu-Ulm, Germany). Photomicrographs were quantified to determine the mean cross-sectional area of cardiomyocytes and interstitial collagen fraction using Leica Qwin V3 computer-assisted image analysis software (Leica Microsystem, Wetzlar, Germany). Average data reflect results from at least four or five different hearts in each group (more than 90 cells for each heart).

Measurement of the BAY 41-8543 concentration in plasma, BAL, and lung tissue samples

The lung tissue was lyophilized and homogenized. Lung tissue, BAL and plasma samples were treated with acetonitril and ammoniumacetate-buffer (0.01 M, pH 6.8). After centrifugation at 1000 g the supernatant was subjected to LC-MS/MS for quantification of BAY 41-8543. Therefore an Agilent 1100 HPLC with a flow rate of 400 µL/min was used. A linear gradient on a Luna 5µ C8(2) 100A 50x2mm (Phenomenex) separation column from 10 to 85 % of acetonitrile with 0.1 % formic acid was performed against a second mobile phase of ammoniumacetate-buffer (0.01 M, pH 6.8). A TurboV ionsource was used to transfer the eluate into an API4000 (AB Sciex) mass spectrometer. The lower limit of quantification was 1 µg/L.

Schermuly, R. T., E. Dony, H. A. Ghofrani, S. Pullamsetti, R. Savai, M. Roth, A. Sydykov, Y. J. Lai, N. Weissmann, W. Seeger, et al. 2005. Reversal of experimental pulmonary hypertension by PDGF inhibition. *J Clin.Invest* 115:2811-2821.

Schermuly, R. T., H. A. Ghofrani, B. Enke, N. Weissmann, F. Grimminger, W. Seeger, C. Schudt, and D. Walmrath. 1999. Low-dose systemic phosphodiesterase inhibitors amplify the pulmonary vasodilatory response to inhaled prostacyclin in experimental pulmonary hypertension. *Am J Respir Crit Care Med* 160:1500-1506.

Schermuly, R. T., S. S. Pullamsetti, G. Kwapiszewska, R. Dumitrascu, X. Tian, N. Weissmann, H. A. Ghofrani, C. Kaulen, T. Dunkern, C. Schudt, et al. 2007. Phosphodiesterase 1 upregulation in pulmonary arterial hypertension: target for reverse-remodeling therapy. *Circulation* 115:2331-2339.

Hardziyenka M., Campian M.E.,. M. de Bruin-Bon R., Michel M.C., Tan H.L. Sequence of Echocardiographic Changes During Development of Right Ventricular Failure in Rat. J Am Soc. Echocardiogr 2006;19:1272-1279.
